# The survival motor neuron protein: structure, functions, stability, and therapeutic targeting

**DOI:** 10.3389/fncel.2026.1894100

**Published:** 2026-08-18

**Authors:** Bradley R. Smith, Rachel Massalee, Mason Mayer, Brendan M. Melvin, Arianna Valeri, Cecelia C. Mangione, Barrington G. Burnett

**Affiliations:** Department of Anatomy, Physiology, and Genetics, Uniformed Services University of the Health Sciences, F. Edward Hebert School of Medicine, Bethesda, MD, United States

**Keywords:** neurodegeneration, post-translational modifications, proteostasis, spinal muscular atrophy, survival motor neuron, ubiquitin-proteasome system

## Abstract

The survival motor neuron (SMN) protein is an essential and highly versatile assembly factor that coordinates RNA metabolism and ribonucleoprotein (RNP) complex formation across multiple cellular compartments. Although SMN is required for the survival of virtually all cell types, its deficiency disproportionately affects *α*-motor neurons, causing their selective degeneration and giving rise to spinal muscular atrophy (SMA). Once viewed primarily as a motor neuron disease, SMA is now understood to be a multi-systemic disorder in which cell-intrinsic dysfunction extends to skeletal muscle, inflammatory glial cells, and metabolic organs. This review examines the regulatory mechanisms that control SMN protein stability, collectively termed proteostasis, with a focus on how post-translational modifications coordinate with the ubiquitin-proteasome system and the autophagy-lysosomal pathway to govern protein turnover and clearance. We also address the emerging concept of gene dosage sensitivity, including the underappreciated paradox that therapeutic SMN overexpression can be as harmful as deficiency, producing distinct toxicities in both neuromuscular and peripheral tissues. Finally, we highlight the need for next-generation combination therapies that integrate genetic modifiers, targeted degradation strategies, and post-translational regulators to maintain SMN levels within the narrow physiological range required for safety and efficacy.

## 1 Introduction

The survival motor neuron (SMN) protein is a 38-kDa, ubiquitously expressed protein that is required for cellular viability in all eukaryotes (Lefebvre et al., 1995). Found in both the cytoplasm and the nucleus, it is best known as the central architect of the spliceosome, operating as a macromolecular assembly factor that drives the construction of ribonucleoprotein (RNP) complexes (Pellizzoni et al., 1998). Despite the ubiquitous presence of SMN and its necessity for basic cellular function, an embryological deficiency in SMN leads to the selective degeneration of lower motor neurons in the spinal cord. Clinically, SMN deficiency causes a disease known as spinal muscular atrophy (SMA). SMA is an autosomal recessive neurodegenerative disorder that is the leading monogenic cause of infant mortality (Lefebvre et al., 1995). SMA presents as progressive muscle weakness and atrophy with widely varying levels of severity, with the most severe form of the disease leading to fatal respiratory failure as young as 2 years old (Talbot and Tizzano, 2017). Remarkably, even though SMA entails significant disease burden, a complete experimental analysis of its full-length protein structure and diverse functional mechanisms has been severely limited. With the rapid evolution of advanced computational models and the recent advent of targeted disease-modifying therapies, a comprehensive and updated synthesis of SMN biology is now both timely and essential.

### 1.1 Genetic context: the *SMN1* and *SMN2* genes

During hominid evolution, an inverted duplication of the *SMN* gene on chromosome 5q13 resulted in two nearly identical paralogs: the telomeric *SMN1* and the centromeric *SMN2* (Lefebvre et al., 1995). Both genes are capable of coding for the exact same protein. However, *SMN2* harbors a critical, translationally synonymous C-to-T point mutation at position +6 in exon 7 (Lorson et al., 1999; Monani et al., 1999). Although this single nucleotide change does not alter the encoded amino acid, it has profound functional consequences: it disrupts an exonic splicing enhancer and simultaneously creates an exonic splicing silencer. Consequently, during the transcription of *SMN2*, exon 7 is skipped in roughly 85 to 90 percent of the resulting mature mRNA transcripts (Lorson et al., 1999). The translation of this abnormally spliced mRNA yields SMNΔ7, a truncated protein lacking its natural C-terminus. In healthy individuals, the *SMN1* gene encodes the full-length protein required for cellular function. However, in patients with SMA, the *SMN1* gene is typically lost due to homozygous deletion or mutation, leaving them entirely reliant on the low levels (10 to 15 percent) of full-length SMN generated by their *SMN2* copies (Lefebvre et al., 1995).

Notably, expression from the *SMN2* gene is highly regulated by epigenetic mechanisms that introduce clinically significant inter-individual variability in SMN protein levels, a key determinant of disease severity and therapeutic response. These mechanisms, including DNA methylation, histone modifications, chromatin remodelling, and non-coding RNA regulation, are essential modulators of *SMN2* expression (Li et al., 2025). Central to this regulation is chromatin accessibility at the *SMN2* expression levels (Li et al., 2025). A compacted chromatin conformation at the *SMN2* promoter region suppresses transcription, whereas an open chromatin conformation increases transcriptional activity, a distinction with direct implications for the epigenetic modulation of SMN2 output as a therapeutic strategy.

The regulatory landscape governing *SMN2* exon 7 splicing is considerably more complex than a single splice-site substitution would suggest. Both cis-acting elements and trans-acting splicing factors that modulate exon 7 inclusion have been catalogued, alongside epigenetic mechanisms including histone acetylation and promoter methylation that impose an additional layer of transcriptional control over *SMN2* output (Virata et al., 2025). This multi-tiered regulatory architecture explains why therapeutic agents targeting the same locus can differ substantially in magnitude and kinetics of SMN upregulation, and underscores the rationale for combining upstream epigenetic modulators with downstream splicing modifiers to maximize *SMN2* productivity.

### 1.2 Physiological significance and selective vulnerability

The central enigma of SMN biology lies in the selective vulnerability of motor neurons. While the low levels of full-length SMN produced by *SMN2* are sufficient to keep most somatic tissues alive, *α*-motor neurons are uniquely sensitive to this deficit. This vulnerability primarily stems from high splicing demands and specialized neuronal functions, such as the axonal transport of localized mRNAs over long distances to maintain the neuromuscular junction (NMJ) (Fallini et al., 2012).

However, even within the central nervous system, not all motor neurons exhibit the same degree of susceptibility. Pathological studies and animal models reveal a distinct gradient of vulnerability (Woschitz et al., 2022). Larger, fast-fatigable motor neurons innervating proximal muscles degenerate rapidly, whereas slow-twitch motor neurons and specific cranial nerve nuclei (such as the oculomotor nucleus) remain highly resistant to SMN depletion (Murray et al., 2008). Interestingly, this differential vulnerability is not strictly correlated with local SMN protein expression levels, as both resistant and vulnerable neuronal pools exhibit similarly diminished SMN concentrations (Murray et al., 2008). Instead, resistance appears to be driven by unique, cell-intrinsic transcriptional adaptations. Laser capture microdissection sequencing (LCM-seq) has shown that resistant motor neurons mount distinct neuroprotective responses, whereas vulnerable pools fall prey to aberrant disease cascades, such as the pathological activation of the p53 apoptotic pathway and the dysregulation of α-synuclein (Kline et al., 2017; Simon et al., 2017).

Understanding these divergent survival mechanisms carries profound therapeutic implications. It suggests that highly vulnerable motor neuron pools possess a much narrower therapeutic window, necessitating extremely early (ideally pre-symptomatic) intervention with SMN-restoring therapies to prevent irreversible synaptic loss (Wirth, 2021). Critically, this also highlights the potential need for SMN-independent neuroprotective agents, such as p53 inhibitors, to support and rescue the most susceptible neurons alongside canonical gene therapies (Simon et al., 2017).

## 2 Protein structure and domain architecture

Understanding the functional versatility of the SMN protein requires a detailed look at its physical architecture. SMN is characterized by specific, highly conserved functional domains interspersed with intrinsically disordered regions. The subcellular distribution of these domains, and the trafficking mechanisms that move SMN between compartments, are intimately linked to its biological roles.

### 2.1 Experimental structural determination and cytoskeletal links

**Figure 1:**
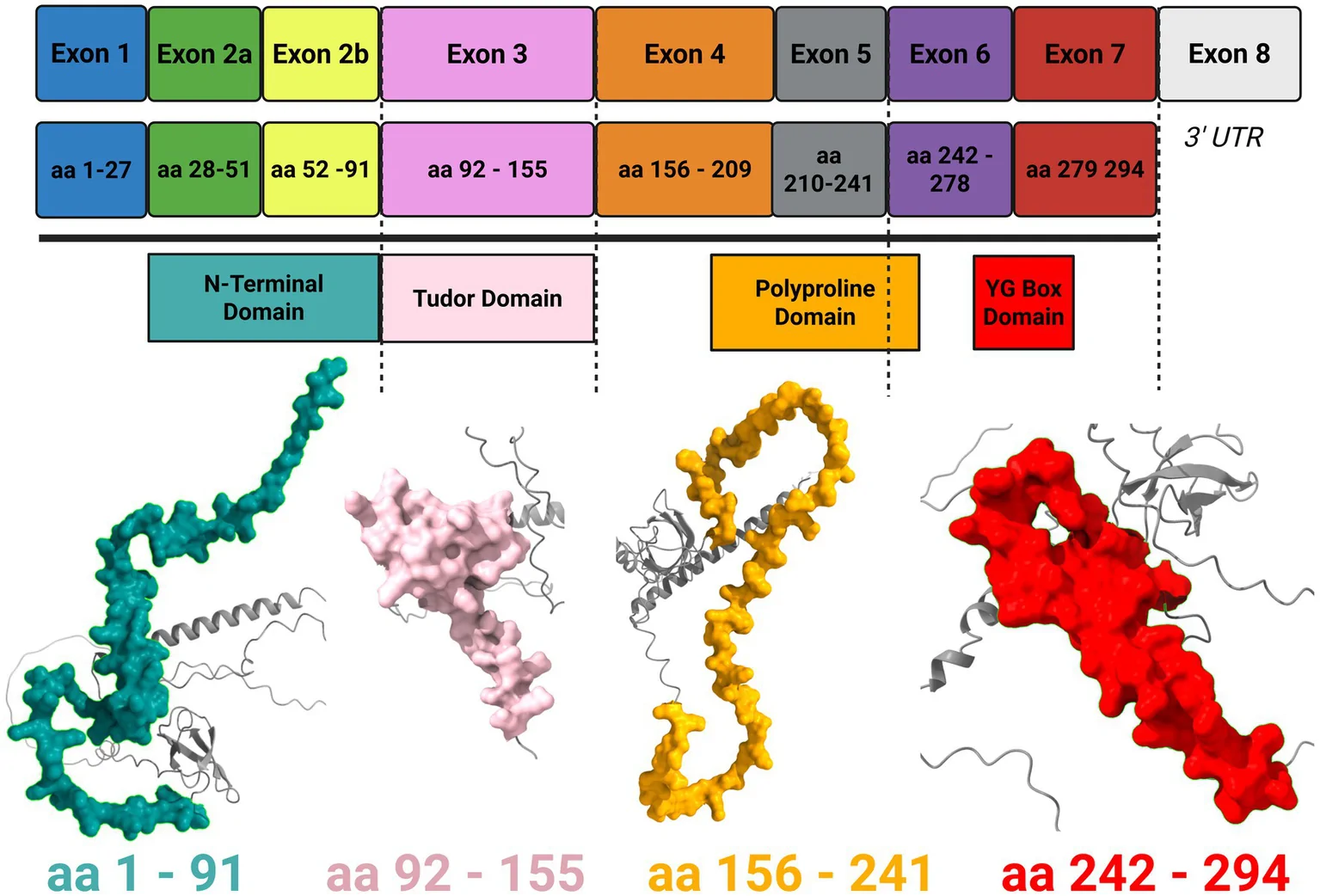
Functional Domain Architecture of SMN. Functional domain architecture of the full-length SMN protein (294 amino acids). The schematic maps the four major functional domains across the nine-exon gene structure: the N-terminal domain (aa 1-91; exons 1-2b), the Tudor domain (aa 92-158; exon 3), the polyproline-rich region (aa 159-241; exons 4-5), and the YG-box domain (aa 242-294; exons 6-7). Exon 8 constitutes the 3’ untranslated region and does not contribute to the protein coding sequence. Functional domain boundaries reflect the locations of true structural motifs rather than strict exon demarcations; for example, the YG-box glycine zipper spans contributions from both exon 6 (aa 242-278) and exon 7 (aa 279-294). Protein structures of isolated functional domains created using AlphaFold 3. Figure created in BioRender. Smith, B. (2026).

The full-length SMN protein consists of 294 amino acids with a molecular weight of 38 kDa. The human *SMN1* and *SMN2* genes encompass 9 exons: exons 1, 2a, 2b, 3, 4, 5, 6, 7, and 8. Exons 1 through 7 encode the 294 amino acids of the functional protein, while exon 8 acts as the 3′ untranslated region (Rimoldi et al., 2025). The protein is divided into four distinct functional zones: the N-terminal domain, the central Tudor domain, the polyproline-rich domain, and the C-terminal YG-box domain, summarized visually in Figure 1. These functional zones can encompass either one or multiple exons, reflecting the differential contribution of one or more exons to the construction of their namesake functional motifs. For example, the true functional polyproline motif is encoded for by subsets of both exons 4 and 5, meaning exons 4 and 5 are classically grouped together as the polyproline-rich domain. Similarly, the YG box domain contains a namesake glycine zipper motif, although only a subset of residues spanning exons 6 and 7 form the true glycine zipper. Domain notations in Figure 1 reflect the locations of true structural motifs. Historically, the full-length SMN protein has evaded complete experimental structure determination. Traditional techniques like X-ray crystallography and nuclear magnetic resonance (NMR) spectroscopy have been limited because highly ordered, defined regions of the protein are interspersed with highly unstructured, flexible domains (Sprangers et al., 2003; Martin et al., 2012). Experimental models are largely restricted to isolated ordered domains, including the N-terminal Gemin2-binding domain, the central Tudor domain, the polyproline-rich region, and the C-terminal YG-box (Martin et al., 2012; Sprangers et al., 2003).

### 2.2 Functional architecture of SMN protein domains

The N-terminal domain of the SMN protein, encompassing amino acids 1 through 91, forms the foundational scaffold required for the assembly, stability, and localization of the entire macromolecular SMN complex. This region is encoded by three discrete exons: exon 1 (amino acids 1–27), exon 2a (amino acids 28–51), and exon 2b (amino acids 52–91). The primary functional role of exon 1 is to act as the N-terminal regulatory interface. While the first 15 amino acids of this region are evolutionarily poorly conserved, the alanine at position 2 (Ala2) is a highly conserved exception, and has the potential to act as the obligate substrate for N-terminal acetylation (Van Damme et al., 2012; Arnesen, 2011). The presence of this specific amino acid sequence is theorized to protect the SMN protein from recognition by the N-end-rule proteasomal degradation pathway, ensuring baseline protein stability before the larger multimeric complex is fully assembled.

Exon 2a of the N-terminal domain forms an amphipathic *α*-helix that embeds into a hydrophobic cavity on Gemin2. The binding of Gemin2 to this specific domain forms a stable SMN-Gemin2 heterodimer, which constitutes the absolute structural core of the SMN complex (Sarachan et al., 2012). Exon 2a initiates a highly conserved basic/lysine-rich domain, and is functionally indispensable for direct, high-affinity interaction of SMN with Gemin2 (Zhang et al., 2011). Without this specific sequence, the SMN protein cannot stabilize or assemble the spliceosomal small nuclear ribonucleoproteins (snRNPs). Beyond its Gemin2-binding role, the sequence encoded by exon 2a is directly responsible for the SMN protein’s intrinsic nucleic acid binding capabilities, which are vital for chaperoning ribonucleoproteins (Yi et al., 2020).

Continuing the basic/lysine-rich structural motif, exon 2b expands the SMN complex’s role beyond spliceosome assembly and into active cytoplasmic transport. Functionally, exon 2b contains crucial dilysine motifs that directly mediate binding to the COPI vesicle coatomer subunit α-COP (Custer et al., 2013). This interaction allows the SMN complex to traffic neuronal processes and is required for normal motor axon growth and neurite outgrowth (Li et al., 2015). Of note, specific residues within this exon govern the liquid–liquid phase separation (LLPS) properties of SMN, allowing the protein to condense and localize into nuclear Cajal bodies (Schilling et al., 2021).

Encoded entirely by exon 3, the central Tudor domain spans amino acids 92 through 158, and adopts a conserved, barrel-like fold comprising five antiparallel β-strands arranged around a hydrophobic core. Although the central Tudor domain is most commonly referenced as residing within amino acids 92–158, only residues 92–144 adopt a well-defined tertiary structure (Sprangers et al., 2003). This highly conserved structural domain is the primary interaction interface for recognizing and recruiting modified partner proteins. It recognizes and binds symmetrically dimethylated arginine (sDMA) residues on target proteins, effectively anchoring SMN to essential client proteins in both the nucleus and cytoplasm (Sprangers et al., 2003; Côté and Richard, 2005).

The polyproline-rich region spans exon 4 (amino acids 159 through 209) and exon 5 (amino acids 210 through 241), and is particularly critical for linking SMN to the actin cytoskeleton by directly binding Profilin2a (Giesemann et al., 1999). This domain is vital for neuronal-specific functions with dynamic cytoskeletal components, including motor axon pathfinding, neurite outgrowth, and endocytosis. SMN deficiency leads to the hyper-activation of the downstream RhoA/ROCK signaling pathway, causing the abnormal hyperphosphorylation of Profilin2a, which subsequently drives an increased F−/G-actin ratio (Schüning et al., 2024). Ultimately, this severe dysregulation of actin dynamics results in neuronal growth cone collapse and impaired synaptic development in SMA patients.

The C-terminal YG-box domain is the structural engine responsible for driving a multitude of macromolecular capabilities exhibited by the SMN protein. The YG-box domain as a whole spans amino acids 242 through 294. Within this region, exon 6 encodes residues 242–278 and exon 7 encodes the final 16 residues, 279–294. The functional designation “YG-box” therefore encompasses both exons, and the glycine zipper motif that defines the domain’s name spans contributions from both. Structural experiments have identified a crystal structure for the isolated YG-box dimer. The dimer is an interdigitated, antiparallel arrangement in which the YG-box domain monomers fold back upon one another to create an extensive symmetric interface stabilized primarily by hydrophobic contacts between the conserved tyrosine and glycine residues that give the domain its name (Martin et al., 2012). This YG-box domain forms a rare example of a soluble and reversible glycine zipper under physiological conditions, facilitating oligomerization of the soluble SMN protein.

### 2.3 Computational modeling and AlphaFold

**Figure 2:**
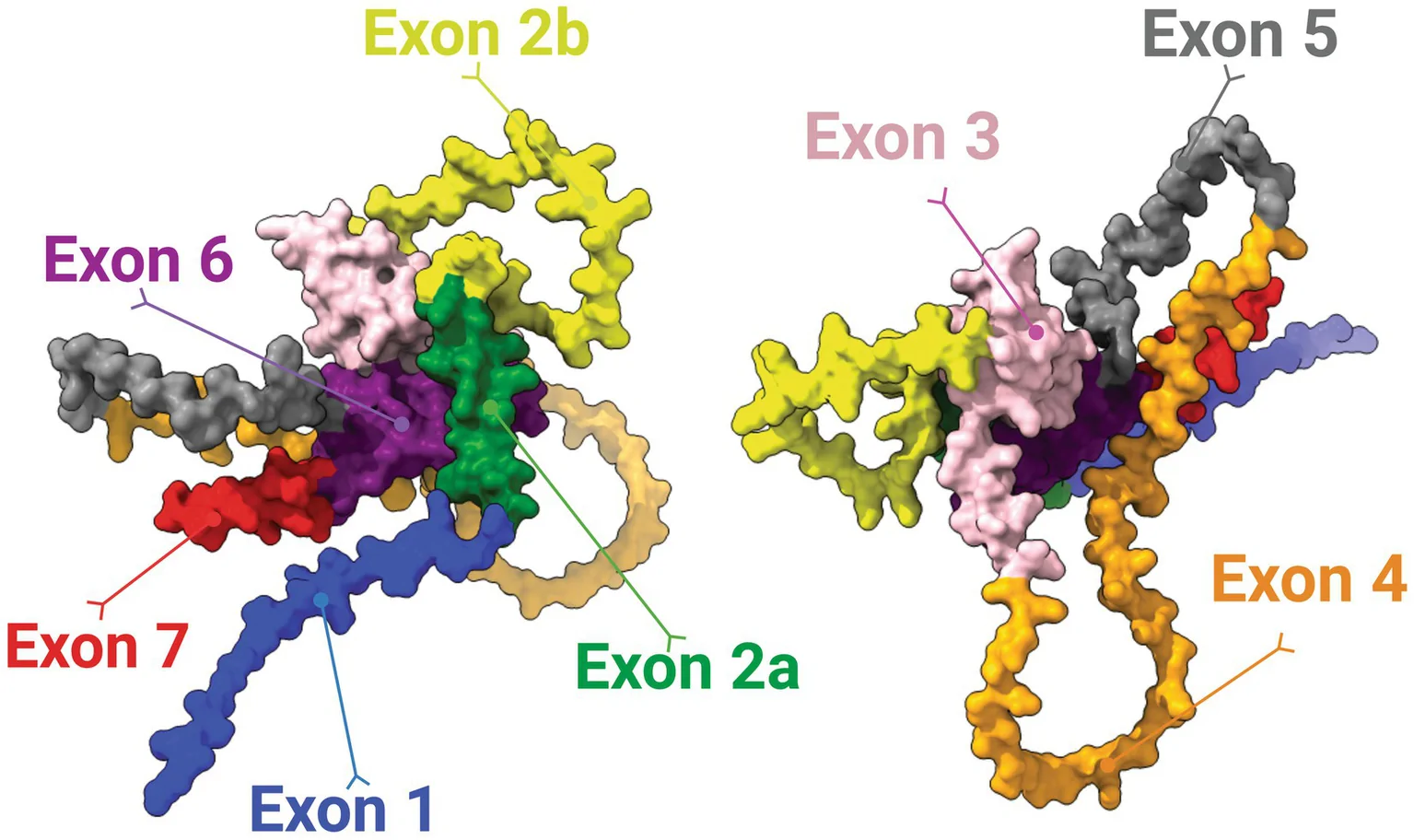
Predicted Structure of Full-Length SMN. AlphaFold 3-predicted structure of full-length, wild-type SMN protein (294 amino acids), annotated by exon contribution. Exon-encoded regions are color. Intrinsically disordered regions, particularly within the polyproline-rich domain (exons 4-5), are predicted with lower local confidence (pLDDT < 70) and are represented as extended or flexible linker segments; these regions have historically resisted experimental structure determination by X-ray crystallography and NMR. The model is presented to illustrate the overall domain topology and relative positioning of structural elements and should be interpreted with this caveat in mind. Figure created in BioRender. Smith, B. (2026).

To fill this significant structural gap, researchers rely heavily on advanced computational modeling. AlphaFold plays a critical role in providing a predicted structural model for full-length SMN (Grimmler et al., 2026). Along with comparative modeling tools like RosettaCM, AlphaFold allows researchers to visualize how the unstructured regions may connect to solved domain structures, aiding in the broader understanding of SMN’s complex molecular interactions. An AlphaFold 3 rendering of full length, wild-type SMN protein is presented in Figure 2. The intrinsic flexibility and instability of the full-length protein have historically confounded traditional crystallization efforts, underscoring the vital importance of these robust computational models to guide ongoing structural investigations. While AlphaFold and isolated domain structures, such as the SMN-Gemin2 core complex solved by NMR, have advanced our understanding, the exact stoichiometry and 3D architecture of the entire megadalton SMN-Gemin multiprotein complex remains a major unsolved challenge in the field.

### 2.4 Mapping SMA-causing missense mutations

**Figure 3:**
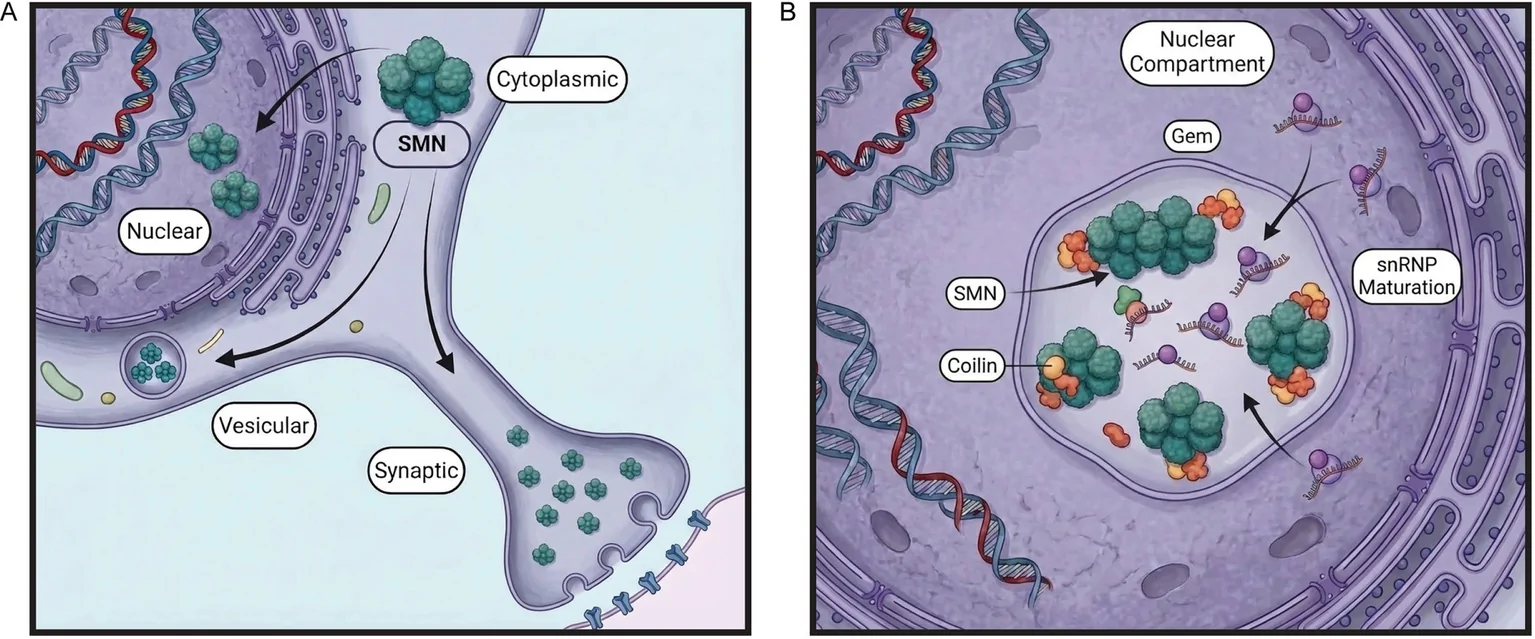
Cellular Distribution of SMN. **(A)** Schematic of SMN protein distribution in the neuronal cytoplasm. SMN exists in the cytoplasm both as a free monomer and assembled into large ribonucleoprotein (RNP) complexes. In motor neurons, SMN associates with RNA-binding proteins including hnRNP R and HuD to form messenger ribonucleoprotein (mRNP) transport granules that travel anterogradely along the microtubule network, delivering localized mRNA transcripts to the distal axon and neuromuscular junction (NMJ). Key structural elements depicted include: SMN complexes (cytoplasm), mRNP granules (axon), and the NMJ terminal (bottom). **(B)** Schematic of SMN protein distribution in the nuclear compartment. SMN concentrates in Gems, discrete nuclear bodies where it colocalizes with coilin to facilitate the final maturation of small nuclear ribonucleoproteins (snRNPs). Cajal bodies and Gems, which may be spatially distinct or overlapping depending on cell type and activity state, are indicated. Figure created by refining a BioRender-designed illustration using Google’s Gemini 3 Flash (Nano Banana 2 model).

The most common disease-causing mutation results in the skipping of exon 7 removes the final 16 amino acids of the SMN protein. Consequently, translation reads into the normally untranslated exon 8, appending a 4-amino-acid degradation signal: EMLA. Lacking the terminal half of the YG-box, the resulting SMNΔ7 protein cannot form glycine zippers to oligomerize. The monomeric state exposes the SCFSlmb degron, and the aberrant EMLA tail, visualized in Figure 3, further accelerates recognition by the ubiquitin-proteasome system, dropping the protein half-life from greater than 8 h to less than 3 h.

While most SMA cases arise from the exon 7 splicing defect, rare spontaneous missense mutations also cause the disease (Polverini et al., 2024; Li, 2017; summarized in Table 1). By mapping these amino acid substitutions onto the full-length 3D AlphaFold model, researchers can visualize how a single mutation might disrupt local hydrogen bonding networks or alter the surface charge of crucial binding interfaces, driving the SMA phenotype. Importantly, catalogued mutations are distributed across all domains of the SMN protein and produce SMA phenotypes of varying clinical severity.

**Table 1:** Summary chart of spinal muscular atrophy-causing mutations by exon.

Mutation category / residue	Exon / domain	Predicted structural biology effect	Clinical SMA phenotype
A2G, A2V, E14X, Q27X	Exon 1 (N-terminus)	Eliminates NatB N-terminal acetylation site; potentially exposes the monomer to N-end-rule proteasomal degradation.	A2G: Type II, III A2V: Type III (Kubo et al., 2015, Monani et al., 2000) E14X: Type I Q27X: Type I (Polverini et al., 2024; Li, 2017)
D30N, D44V	Exon 2a (Gemin2 Binding)	Perturbs the structural interface for Gemin2 binding, decoupling the core stabilization required for the fundamental heterodimeric building block of the complex.	D30N: Type II D44V: Type III (Sun et al., 2005)
S63X	Exon 2b	Alters physical confirmations of sequences proximal to functional motifs which mediate binding to *α*-COP and mTOR-dependent phase separation.	S63X: Type I (Wijaya et al., 2021)
W92S, V94F, V94G, G95R, Y109C, W102X, A111G, I116F, Y130C, Y130H, E134K, Q136E, L141P, Q154X, Q157X	Exon 3 (Central Tudor Domain)	Physically collapses or distorts the folded reader module. Abolishes interaction with symmetrically dimethylated arginine (sDMA) ligands on Sm proteins. G95R and I116F specifically cause temperature-sensitive misfolding.	Type I: W92S, V94F, A111G, I116F, Q136E, L141P Type I, II: E134K Type II: V94G, Q157X Type III: G95R, Y109C, Y130C, Y130H, Q154X (Cuscó et al., 2004; Wirth, 2000; Clermont et al., 2004; Fraidakis et al., 2012; Wijaya et al., 2021; Prior, 2007; Mendonça et al., 2020)
D181G, A188S, W190X, P221L, L228X, S230L	Exon 4 & 5 (Polyproline-Rich)	Alters the unstructured repeating proline architecture, diminishing physical docking with profilin and thereby affecting the protein’s linkage to cellular actin dynamics.	Type I: A188S, P221L, W190X Type II, III: S230L N/A (Unclassified): D181G (Alías et al., 2009; Zapletalová et al., 2007)
P244L, P245L, L260S, S262G, S262I, M263R, M263T, S266P, M269T, Y272C, H273R, T274I, G275S, Y276H, Y277C, G279V, G279D, F280I, R288M, R288X	Exons 6, 7 & 8 (C-Terminal YG Box)	Disrupts the helical glycine zipper required for SMN self-oligomerization. Traps the protein as a monomer, sterically exposing an internal F-box phosphodegron that signals for SCF-Slmb-mediated rapid degradation.	Type I: M263R, Y276H, G279V Type I, II: R288M Type I, II, III: Y272CType II: L260S, M263T, S266P, H273R Type II, III: T274I, Y277C Type III: P244L, P245L, S262G, S262I, M269T, G275S N/A (Unclassified): G279D, F280I (Butchbach, 2021; Talbot et al., 1997; Sharifi et al., 2021; He et al., 2013)
G279C	Exon 7 (YG Box)	Induces an abnormal, hyper-oligomeric state where the protein self-associates too strongly and binds excess levels of Gemins 3, 4, and 5.	Type II, III (Martin et al., 2012)
K119R (Experimental)	Exon 3 (Tudor Domain)	Replaces target lysine for CBP-mediated acetylation; redirects SMN into PML microbodies and modifies Cajal body dynamics.	N/A: Experimental Cell Line Mutant (Lafarga et al., 2018)
S290D (Experimental)	C-Terminus	Phospho-mimetic addition of negative charge drastically reduces protein stability and suppresses self-oligomerization.	N/A Experimental Cell Line Mutant (Detering et al., 2022)
S139S	Exon 3 (Tudor Domain)	Translationally silent; alters mRNA structural architecture/splicing without changing the final folded protein sequence.	N/A (Unclassified, Butchbach, 2021)
Nonsense Mutations (E14X, Q15X, Q27X, S63X, W102X, Q154X, Q157X, W190X, L228X, Q282X, R288X)	Throughout	Generates severely truncated, misfolded polypeptides lacking the essential C-terminal oligomerization engine and Tudor domain (if early enough), forcing rapid degradation.	Type I: E14X, Q27X, S63X, W190X, L228X, Q282X Type I, II, III: Q15X Type II: Q157X, R288X Type II, III: W102X Type III: Q154X (Butchbach, 2021)
Frameshift Indels (c.19delG, c.22dupA, c.430del4, etc.)	Throughout	Distorts reading frame, resulting in premature stop codons. Causes massive structural loss of downstream domains (Tudor, Proline, YG box), producing unstable, non-functional monomers.	Varies widely by exact mutation, spanning the entire spectrum. Examples: Type I: c.19delG, c.401_402delAG, c.439_443del Type I, II, III: c.22dupA, c.430del4 Type II: c.56delT, c.542delGT Type III: c.-7-9del, c.773insC (Butchbach, 2021)
Splice-Site Alterations (c.835-2A > G, c.835-5 T > G, etc.)	Exon/Intron Boundaries	Induces aberrant splicing (e.g., Exon 7 skipping to produce SMNΔ7, removing the terminal 16 amino acids of the YG box. Abrogates glycine zipper formation, exposing the internal degron).	Mostly Type I, but variable. Examples: Type I: c.*3 + 3A > T, c.834 + 2 T > G, c.835-2A > G, c.835-3C > A, c.835-5 T > G, c.867 + 2 T > G Type III: c.835-1G > A, c.922 + 6 T > G (Butchbach, 2021)

SMA, spinal muscular atrophy. Mutation nomenclature follows standard amino acid single-letter codes. Phenotypes classified according to SMA type (I–III) based on reported clinical presentation in the literature.

## 3 Liquid–liquid phase separation and subcellular localization

### 3.1 Liquid–liquid phase separation and stress granule dynamics

The SMN complex acts as a master assembly chaperone for RNP complexes that partition into membraneless organelles, or biomolecular condensates (BMCs), such as Cajal bodies, Gemini of Cajal bodies (Gems), and stress granules (Ham and Lee, 2026; Han et al., 2024). These condensates regulate cellular biochemistry by creating liquid-like droplets where RNA and proteins are locally concentrated, reflecting a property known as liquid–liquid phase separation (LLPS). SMN-mediated phase separation relies on its central Tudor domain which specifically reads sDMA modifications on protein ligands, effectively coordinating SMN phase separation and condensation onto independent modified partner proteins across cellular compartments (Courchaine et al., 2021). A critical feature of LLPS behavior is that the formation of BMCs by constituent proteins is fundamentally driven by the thermodynamics of protein concentration. In the context of SMN LLPS, phase separation is dependent on the local concentration of the SMN protein and is uniquely modified by SMN post-translational modifications (Mukherjee et al., 2023; Schilling et al., 2021; Li et al., 2022).

Of emerging interest in SMA biology is the relationship between SMN and stress granules. SMN deficiency drastically reduces the ability of mammalian cells to form stress granules in response to stress, although stress granule formation in these cells can be rescued by the overexpression of G3BP1 (Zou et al., 2011). Of additional interest, SMN is shown to localize to the plasma membrane and interact with Caveolin 1, a ubiquitously expressed regulator of lipid raft formation (Gabanella et al., 2016). As BMC formation in Cajal bodies and Gems reflects a homeostatic coordination between compartmental SMN concentrations and PTM state, stress granule formation represents an underexplored aspect of SMN LLPS behavior that requires further investigation.

### 3.2 Cellular distribution of SMN

**Figure 4:**
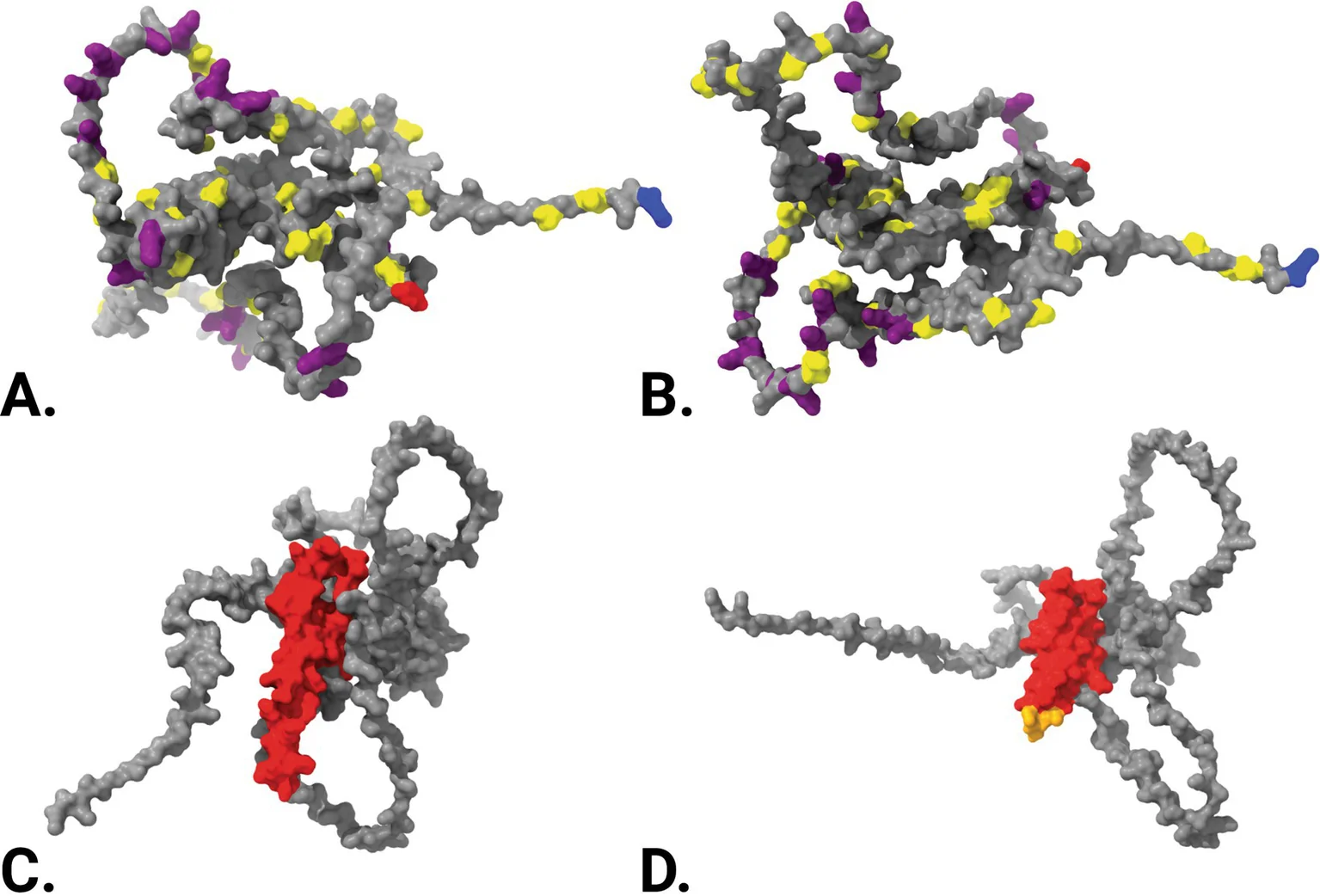
Post-translational modification (PTM) site mapping and C-terminal structural comparison of WT and truncated SMN isoforms. **(A)** Anterior view of predicted full-length WT SMN protein showing all candidate lysine residues (purple) as potential K-linked ubiquitination sites and all serine, threonine, and tyrosine residues (yellow) as potential phosphorylation sites. The N-terminus is colored blue and the C-terminus red. **(B)** Posterior view of the same structure, providing a complete survey of PTM site distribution. Note that experimentally validated sites represent a subset of the candidate residues shown. **(C)** WT SMN protein with the YG-box domain highlighted in red, illustrating the intact C-terminal oligomerization interface. **(D)** Predicted structure of SMNΔ7, the truncated isoform produced by SMN2 exon 7 skipping. The abbreviated YG-box is highlighted in red, and the aberrant four-amino-acid EMLA tail, a degron created by read-through into exon 8, is highlighted in orange. Loss of the terminal YG-box half destabilizes oligomerization and exposes an internal SCFSlmb phosphodegron, accelerating proteasomal degradation. Protein structures generated with AlphaFold 3. Figure created in BioRender. Smith, B. (2026).

The subcellular localization of the SMN protein reflects its highly dynamic and pleiotropic nature, and is tightly regulated by the post-translational modifications described in Section 5. In the cytoplasmic compartment, the SMN protein exists both in a free state and as part of massive multiprotein complexes, as visualized in Figure 4A, allowing for the initial loading of Sm proteins onto newly transcribed small nuclear RNAs (snRNAs) (Paushkin et al., 2002). Beyond the cell body, SMN exhibits specialized transport mechanisms in highly polarized cells like motor neurons. In these cells, SMN forms messenger ribonucleoprotein (mRNP) transport granules that actively travel along the microtubule network. This axonal transport machinery is vital for delivering localized mRNA transcripts directly to the distal neuromuscular junction (Setola et al., 2007; Rossoll et al., 2003). The biogenesis of snRNPs is inherently reliant on massive inter-compartmental transport. After cytoplasmic assembly, the SMN complex recruits importin adaptors to facilitate nuclear import of itself and its constituent components, enabling the dynamic nuclear activities required for spliceosome maturation (Narayanan et al., 2002). Within the nuclear compartment, SMN heavily concentrates in Gems (Liu and Dreyfuss, 1996). In these discrete nuclear domains, SMN frequently colocalizes with coilin to facilitate the final maturation of snRNPs, as visualized in Figure 4B.

## 4 Canonical and non-canonical functions of SMN

### 4.1 snRNP biogenesis and the splicing paradox

Within the cytoplasm, newly transcribed snRNAs must be packaged with a heptameric ring of Sm proteins to form functional snRNPs (Pellizzoni et al., 1998; Grimmler et al., 2026). The SMN complex acts as a highly specific assembly factor in this process. The Tudor domain of SMN binds to sDMA motifs on the tails of Sm proteins, loading them onto the snRNA molecule. Once assembled, the complex is imported into the nucleus, localizing to Cajal bodies for final spliceosome maturation (Renvoisé et al., 2006). While this canonical role is universally required for all cells, the most striking clinical findings of SMN deficiency reflect a comparatively rapid degeneration of motor neurons during development. This is frequently attributed to a splicing paradox, wherein low SMN levels preferentially disrupt the assembly of the U12-dependent minor spliceosome. This failure leads to the catastrophic mis-splicing of specific, highly demanded motor neuron transcripts, most notably the crucial synaptic gene *Stasimon* (Lotti et al., 2012; Osman et al., 2020; Simon et al., 2019).

Despite the compelling evidence from the U12 spliceosome and Stasimon literature, the splicing hypothesis remains an incomplete explanation for the selective vulnerability of motor neurons. Several lines of evidence point to RNA-independent deficits as necessary co-contributors. SMN deficiency impairs axonal transport of beta-actin mRNA and associated mRNP granules, disrupts retrograde signaling, and compromises the integrity of the neuromuscular junction independently of splicing dysfunction (Fallini et al., 2012; Rossoll et al., 2003). Additionally, SMN-deficient neurons exhibit impaired mitochondrial membrane potential, elevated reactive oxygen species, and reduced oxidative phosphorylation, creating bioenergetic vulnerabilities that are not directly attributable to altered splicing of any single transcript (Zilio et al., 2022). Finally, microglia-dependent complement-mediated stripping of proprioceptive synapses from motor neurons occurs early in disease, prior to substantial cell death, and represents a synaptic pathology mechanism that operates in parallel to, rather than downstream of, splicing defects (Vukojicic et al., 2019). Taken together, these findings indicate that while the minor spliceosome hypothesis identifies an important contributing mechanism, motor neuron vulnerability likely reflects the convergence of multiple cell-intrinsic and non-cell-autonomous pathways, none of which is individually sufficient. This has practical therapeutic implications, since restoring SMN levels may not fully rescue established synaptic and mitochondrial deficits.

### 4.2 U7 snRNP and histone mRNA processing

Beyond the major and minor spliceosomes, the SMN complex is essential for the assembly of the U7 snRNP (Tisdale et al., 2013). This specific ribonucleoprotein does not participate in pre-mRNA splicing but is instead critical for the 3′-end processing of replication-dependent histone mRNAs. In the context of SMN deficiency, the failure to properly assemble U7 snRNPs leads to the accumulation of aberrantly polyadenylated histone transcripts, severely altering histone gene expression and impacting chromatin dynamics (Tisdale et al., 2013).

### 4.3 Transcription regulation and R-loop resolution

Emerging evidence has expanded the nuclear role of SMN to include the direct maintenance of genomic stability. During transcription, newly synthesized RNA can occasionally hybridize with the DNA template to form abnormal RNA–DNA hybrids known as R-loops. SMN interacts directly with the C-terminal domain of RNA Polymerase II to form the SMN-RNAPII complex, which also assembles with the RNA helicase Senataxin (SETX) to facilitate the resolution of these R-loops. A reduction in SMN levels impairs this clearance process, linking SMA pathology to increased DNA damage and subsequent genomic instability in vulnerable motor neurons (Zhao et al., 2016). R-loop clearance requires SMN to localize to active transcription factories and form competent SMN-RNAPII complexes in the nucleoplasm. ZPR1, a vital cofactor in this complex, functions via ligand-dependent nucleocytoplasmic shuttling; it interacts with cytoplasmic EGF receptors, and upon signaling, releases and translocates into the nucleus to associate with the SMN-RNAPII complex (Gangwani et al., 2001).

### 4.4 Summary of key SMN interactors

SMN’s diverse functional repertoire is dictated by its varied binding partners (Table 2).

**Table 2:** Summary of Key SMN Interactors.

Interacting protein	SMN binding region	Primary cellular consequence
Gemin2	N-terminal Domain	Nucleates and stabilizes the core SMN complex
SmD1, SmD3, SmB	Tudor Domain	Facilitates snRNP biogenesis and splicing regulation
Coilin	Tudor Domain	Localizes the complex to nuclear Cajal bodies
Profilin2a	Proline-Rich Region	Regulates actin cytoskeleton at the synapse
hnRNP R, HuD	Central Region	Facilitates axonal transport and local mRNA translation
Senataxin (SETX)	C-Terminal Complexed (Nuclear)	Resolves R-loops and prevents DNA damage

SMN, survival motor neuron; snRNP, small nuclear ribonucleoprotein; hnRNP, heterogeneous nuclear ribonucleoprotein; SETX, senataxin.

### 4.5 Cytoplasmic and synaptic roles (non-canonical functions)

Maintaining the distal synapse requires massive localized protein synthesis. In the cytoplasm, SMN associates with RNA-binding proteins (e.g., hnRNP R, HuD) to package specific mRNAs into messenger ribonucleoprotein (mRNP) transport granules. These granules travel down the motor axon along microtubules to deliver mRNA, coding for proteins for β-actin, directly to the NMJ (Rossoll et al., 2003; Fallini et al., 2012). SMN’s interaction with Profilin1 and Profilin2a at the presynaptic terminal is also indispensable for maintaining actin cytoskeletal integrity. As described in Section 2.2, SMN deficiency hyper-activates the RhoA/ROCK pathway, causing aberrant hyperphosphorylation of Profilin2a that ultimately drives growth cone collapse and synaptic dysfunction (Schüning et al., 2024).

### 4.6 Vesicular trafficking and endocytosis

SMN is deeply integrated into cellular vesicular trafficking networks. Beyond cytoskeletal regulation, SMN plays an essential role in vesicular trafficking and endocytosis through its interaction with Neurochondrin (NCDN). Neurochondrin is a vital neural protein that localizes specifically to Rab5-positive endosomal vesicles, where it maintains cell polarity and drives neurite and dendrite morphogenesis (Thompson et al., 2018). Decreased function of the SMN protein directly impairs endocytic pathways, contributing significantly to the synaptic dysfunction and transmission failures observed in SMA (Dimitriadi et al., 2016). SMN also interacts with the coatomer protein *α*-COP, localizing to COPI vesicles. Consequently, the depletion of SMN impairs additional critical endosomal trafficking pathways and synaptic vesicle release, further compromising NMJ integrity. Finally, SMN plays an indispensable role in mitochondrial bioenergetics. SMN-deficient cells exhibit reduced mitochondrial membrane potential, impaired oxidative phosphorylation, and elevated reactive oxygen species (ROS), rendering motor neurons uniquely vulnerable to bioenergetic failure and oxidative stress. Mechanistically, SMN loss disrupts the splicing of transcripts encoding mitochondrial respiratory chain components, suggesting that the canonical snRNP assembly function of SMN directly underpins mitochondrial health (Zilio et al., 2022).

## 5 Proteomics and post-translational modifications (PTMs)

The multifaceted functions, subcellular localization, and overall stability of the SMN protein are tightly regulated by a diverse landscape of post-translational modifications (PTMs). These modifications do not operate in isolation; rather, they form an integrated regulatory code that links the functions of SMN to the protein stability mechanisms.

### 5.1 SMN interaction partners

The diverse binding partners catalogued in Table 2 (Section 4.4) reflect only the core interactome. The full SMN interaction network is substantially broader and directly relevant to the post-translational regulation covered in this section. Within the nucleus, SMN associates with RNA Polymerase II, Senataxin (SETX), ZPR1, and nucleolar proteins including Fibrillarin and Nucleolin, which are required for ribosome biogenesis (Pánek et al., 2023; Terns and Terns, 2001; Pellizzoni et al., 2001). SMN also acts as a translation hub by binding ribosomal proteins, eukaryotic initiation and elongation factors, and transcriptional regulators including p53, mSin3A, and the Ewing’s sarcoma protein (Pellizzoni et al., 2001; Young et al., 2003). In the cytoplasm, SMN interfaces with RNA-binding proteins including hnRNP R, hnRNP Q, HuD, IMP1, KSRP, FMRP, FUS, and TDP-43 to mediate axonal mRNA transport, and its nucleocytoplasmic shuttling depends on Importin β and Snurportin1 (Faravelli et al., 2023; Cacciottolo et al., 2019). Critically for the sections that follow, the stability, localisation, and functional capacity of SMN are dynamically governed by direct interactions with the proteostasis network, encompassing the Hsc70 folding chaperone, modifying kinases, the acetyltransferase CBP, and ubiquitin-pathway enzymes from UBA1 through to specific E3 ligases and deubiquitinases (Matera et al., 2024; Kwon et al., 2013). It is within this proteostatic context that post-translational modifications exert their regulatory influence.

### 5.2 SMN phosphorylation sites

The SMN protein contains 50 reported phosphorylation sites, of which at least 26 have been experimentally validated by mass spectrometry, spanning the entire protein but showing a mild enrichment in the N-terminal region (Grimmler et al., 2005). A large cluster of these sites, including S4, S5, S8, S18, S25, S28, S31, T37, Y43, S49, T68, T69, S80, T85, S88, S166, S175, S180, S187, and S192, are primarily targeted by protein kinase A (PKA) to regulate SMN’s interaction with Gemin proteins and coilin, with S28 and S31 being specifically critical for spliceosomal snRNP assembly (Wu et al., 2011). Because SMN is significantly shielded from degradation when successfully integrated into this multiprotein architecture, PKA-mediated phosphorylation acts as a direct, upstream inhibitor of SMN turnover (Wu et al., 2011).

Phosphorylation also dictates the phase separation and subcellular localization of the complex. Specifically, the phosphorylation of serines S49 and S63 drives the condensation of SMN into nuclear Cajal bodies, while the phosphorylation of specific tyrosine residues within the central Tudor domain (Y109, Y127, and Y130) is absolutely required for Cajal body accumulation and nucleocytoplasmic trafficking (Schilling et al., 2021). Finally, at the extreme C-terminus, the phosphorylation of serines S290 and S292 directly governs the protein’s overall stability and its vital capacity for self-oligomerization (Detering et al., 2022). This dynamic phosphorylation landscape dictates the affinity of the SMN complex for its various RNA and protein substrates, ensuring that snRNP assembly occurs efficiently and that the assembled complexes are correctly targeted for nuclear import (Eggert et al., 2006).

### 5.3 SMN methylation and ubiquitination sites

Beyond phosphorylation, the covalent attachment of ubiquitin and ubiquitin-like modifiers represents the primary mechanism dictating SMN lifespan and localization (see Section 6 for the full degradation machinery). Ubiquitination tags monomeric or misfolded SMN for rapid destruction via the ubiquitin-proteasome system (Burnett et al., 2009), operating through a dense network of at least 10 proteomically identified lysine residues: K41, K45, K51, K55, K83, K93, K179, K184, K186, and K209 (Danielsen et al., 2011). Modification at these sites generally acts as a degradation signal dictating cellular half-life. Within this group, lysine 55 (K55) functions as a competitive crosstalk node that can be modified by either ubiquitin or a Small Ubiquitin-like Modifier (SUMO). Beyond canonical polyubiquitination, monoubiquitination also occurs; specifically, the nuclear E3 ligase Itch monoubiquitinates SMN to promote its cytoplasmic retention, thereby regulating the nuclear-cytoplasmic distribution of the protein.

In parallel to this degradation signal, sumoylation and acetylation provide additional layers of spatial regulation (Lafarga et al., 2018). These specific modifications dynamically influence the nuclear-cytoplasmic shuttling of SMN, facilitating its targeted integration into nuclear Cajal bodies where final spliceosome maturation occurs (Han et al., 2016). Methylation dynamics are also indispensable for SMN function. The Tudor domain specifically recognizes sDMA residues on the tails of target Sm proteins (Sprangers et al., 2003). The direct interaction between the SMN Tudor domain and these methylated Sm proteins provides the necessary specificity to efficiently assemble snRNAs, highlighting how both the modification of SMN and its ability to read the modifications of interacting partners are required for cellular survival. A comprehensive overview of candidate SMN ubiquitination and phosphorylation sites are presented in Figure 3, as well as a depiction of SMNΔ7 mutation-induced degron uncovering.

## 6 Protein stability, degradation, and Proteostasis

The steady-state level of SMN is a delicate balance between synthesis and degradation (Burnett et al., 2009). Seminal research established that the intracellular turnover of the SMN protein is primarily mediated by the ubiquitin-proteasome pathway (Chang et al., 2004). Because it lacks the crucial YG-box domain required for self-oligomerization, the SMNΔ7 isoform produced by *SMN2* is highly unstable, making it a prime target for rapid cellular clearance (Locatelli et al., 2015).

### 6.1 The degradation machinery (E3 ligases)

Multiple E3 ubiquitin ligase networks converge to tag SMN for destruction. Mind bomb 1 (Mib1) and Neuralized-like protein 2 (Neurl2) are two essential E3 ubiquitin ligases that work cooperatively to ubiquitinate and promote the proteasomal degradation of SMN (Kwon et al., 2013). They physically interact with the SMN complex and facilitate the attachment of polyubiquitin chains, which serves as a highly specific signal for 26S proteasome degradation. A second critical regulatory mechanism involves a conserved phosphodegron recognised by the SCF Slmb E3 ubiquitin ligase complex and its mammalian ortholog β-TrCP (Gray et al., 2018). When specific serine residues on SMN are phosphorylated, this complex binds the protein and initiates targeted ubiquitination, accelerating its turnover (Gray et al., 2018). Moreover, the truncation of SMNΔ7 exposes a specific degradation signal, or degron, at its altered C-terminus (Cho and Dreyfuss, 2010). This exposed degron makes the truncated isoform an exceptionally vulnerable substrate for the cooperative actions of Mib1, Neurl2, and other E3 ligases.

### 6.2 The stabilization machinery (Deubiquitinases)

Working in direct opposition to the degradation machinery, ubiquitin-specific peptidase 9 X-linked (USP9X) is a deubiquitinating enzyme that cleaves polyubiquitin chains from SMN. By stripping these tags placed by enzymes like Mib1 and Neurl2, USP9X rescues SMN from proteasomal degradation and extends its functional half-life to restore SMN protein levels (Han et al., 2012).

### 6.3 The interplay of UPS and autophagy in neuronal Proteostasis

While the ubiquitin-proteasome system serves as the primary executioner for the rapid turnover of monomeric and misfolded SMNΔ7 fragments, the autophagy-lysosomal pathway provides a distinct and equally critical mechanism for SMN regulation, particularly in post-mitotic motor neurons. Neurons face unique spatial challenges regarding proteostasis. The ubiquitin-proteasome system largely manages localized, immediate protein quality control. In contrast, autophagy is responsible for clearing larger macromolecular SMN complexes and preventing the accumulation of insoluble aggregates, often requiring the retrograde transport of autophagosomes from the distal synapse back to the neuronal soma (Chaytow et al., 2018).

The interplay between these two systems becomes highly relevant in the context of spinal muscular atrophy. SMN physically interacts with the selective autophagy receptor p62 (SQSTM1), indicating that a subset of the SMN protein pool is actively routed toward autophagic degradation (Rodriguez-Muela et al., 2018). When the ubiquitin-proteasome system is overwhelmed, or when therapies like AAV9 induce massive SMN overexpression, cells may rely heavily on autophagy as a compensatory clearance mechanism. Conversely, because SMN actively regulates transcription factor EB (TFEB), SMN deficiency impairs autophagic flux in part through effects on TFEB, the master transcriptional regulator of lysosomal biogenesis and autophagy gene expression. Evidence from motoneuron-like cell lines indicates that SMN loss reduces TFEB expression at the transcript level, suggesting that the regulation is at least partly transcriptional rather than purely post-translational (Ando et al., 2020). Critically, SMN-deficient cells show a concurrent reduction in lysosomal number and activity, consistent with insufficient TFEB-driven transcription of lysosomal pathway genes. Whether SMN acts on TFEB directly through its role in RNA processing and snRNP biogenesis, or indirectly through upstream signaling cascades such as mTORC1 inactivation that normally promotes TFEB nuclear translocation, remains to be definitively established. This distinction carries therapeutic relevance: if SMN regulates TFEB transcriptionally, splicing modifiers may partially rescue lysosomal function; if the link is indirect, more targeted interventions such as mTORC1 inhibition or direct TFEB activators may be required as adjunct strategies (Custer and Androphy, 2014; Ando et al., 2020). This creates a vicious pathogenic cycle where low SMN levels lead to defective autophagy, which in turn causes a global breakdown in neuronal proteostasis, further exacerbating the motor neuron’s inability to clear toxic proteins. Disrupting this cycle by modulating specific autophagic pathways, such as blocking the p62-dependent degradation of SMN, offers a promising therapeutic strategy to stabilize residual SMN levels in patients (Rodriguez-Muela et al., 2018).

## 7 Beyond the motor neuron: cell-autonomous defects in muscle, glia, and metabolic organs

**Figure 5:**
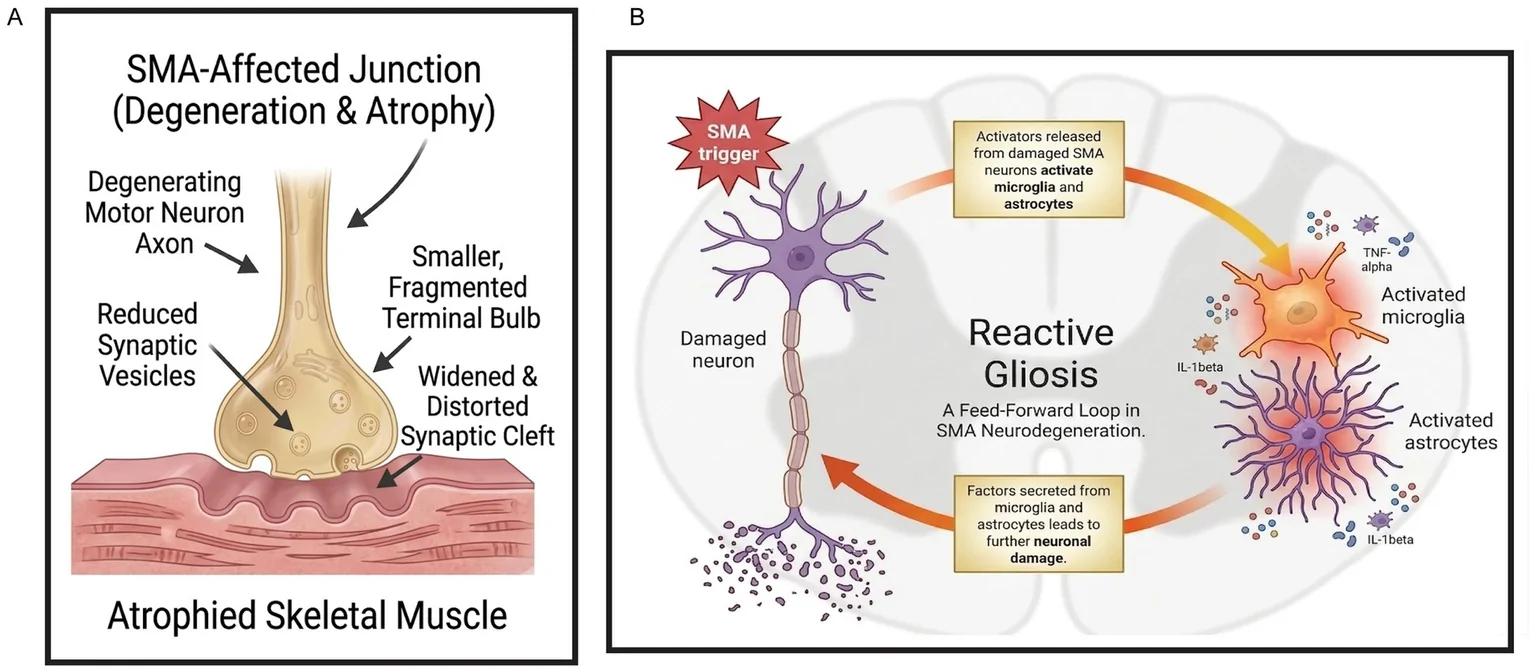
SMA-associated neuropathology at the neuromuscular junction and in glial cells. **(A)** Schematic of an SMA-affected neuromuscular junction (NMJ). The SMA NMJ is characterized by motor neuron axon degeneration, reduced synaptic vesicle density, fragmentation of the presynaptic terminal bouton, and widening and distortion of the synaptic cleft, reflecting impaired vesicular release and NMJ maintenance.**(B)** Schematic of reactive gliosis in SMA, highlighting neuroinflammatory loops in which damage to the neurons triggers microglial and astrocyte activation, leading to the release of cytokines and further neuronal damage. Illustrations created by refining a BioRender-sourced NMJ template using Google’s Gemini 3 Flash (Nano Banana 2 model).

SMN deficiency causes numerous pathological changes in motor neurons, as detailed above and summarized in Figure 5A. Accumulating evidence also demonstrates that SMN deficiency causes profound, cell-autonomous defects in multiple cell types beyond motor neurons, establishing SMA as a genuinely multi-systemic disorder.

### 7.1 Skeletal muscle pathology

While muscle atrophy in SMA is heavily driven by denervation, skeletal muscle is more than a passive bystander. SMN deficiency intrinsically impairs muscle development, maintenance, and structural integrity (Boyer et al., 2013). SMN-deficient myoblasts exhibit severe impairments in fusion and myotube formation due to altered myogenic gene expression, including reduced MyoD, Myogenin, Myomaker, and Myomixer (Bricceno et al., 2014; McCormack et al., 2021). Moreover, SMN plays a direct role in muscle cell maturation via the activation of Caspase-3 and the Akt signaling pathway. Its depletion delays the expression of mature myosin heavy chain (MHC) and drastically reduces the ability of myoblasts to form multinucleated myotubes (Ando et al., 2020).

Structurally, the SMN complex localizes to the sarcomeric Z-disc, where it co-localizes with *α*-actinin (Walker et al., 2008). In this compartment, SMN serves as a direct proteolytic target of calcium-activated calpains, and its deficiency leads to morphological defects consistent with Z-disc disruption (Walker et al., 2008). Beyond sarcomeric disruption, SMN deficiency also disrupts calcium homeostasis by downregulating SERCA2 expression, leading to severe intracellular calcium signaling defects in muscle tissue (Khayrullina et al., 2020). Beyond broader cytoskeletal disruption, SMN deficiency significantly impairs focal adhesion dynamics within skeletal muscle. Focal adhesions are crucial macromolecular assemblies that mechanically link the intracellular actin cytoskeleton to the extracellular matrix. Research demonstrates that SMN-deficient myoblasts exhibit abnormal focal adhesion maturation and turnover, driven primarily by the delayed cleavage of the core focal adhesion protein, Talin (Bricceno et al., 2014). Because the timely remodeling of focal adhesions is an absolute requirement for cell motility and differentiation, this disruption directly contributes to the severe impairments in myoblast fusion and myotube formation observed in SMA, cementing that muscle pathology is a primary cell-autonomous defect rather than solely a secondary consequence of denervation (Bricceno et al., 2014).

### 7.2 Glial cell toxicity and neuroinflammation

Crucially, glial reactivity in SMA is a direct, cell-autonomous consequence of SMN deficiency within the glia themselves, rather than merely a secondary reaction to dying motor neurons, as summarized in Figure 5B. SMN is robustly expressed in wild-type astrocytes and microglia, where its canonical role in RNA splicing tightly regulates the expression of transcripts essential for cellular homeostasis and immune responses (Tapken et al., 2024). In astrocytes, the loss of SMN disrupts intracellular calcium regulation and reduces the secretion of essential neurotrophic factors, pushing the cells into a pro-inflammatory, neurotoxic state (McGivern et al., 2013). This intrinsic astrocytic defect impairs vital cross-talk with motor neurons. Astrocytes derived from SMA induced pluripotent stem cells (iPSCs) exhibit toxic properties that directly induce motor neuron death (Allison et al., 2022). Strikingly, the viral-mediated knockdown of the transcription factor GATA6 specifically within SMA astrocytes prevents this motor neuron loss, highlighting a direct, pathogenic glial-neuronal interaction (Allison et al., 2022).

Similarly, SMN deficiency in microglia fundamentally alters their transcriptome, priming them for hyper-reactivity, increased cellular motility, and enhanced phagocytic activity (Khayrullina et al., 2022). Recent work has redefined the role of these glial cells in SMA by demonstrating their direct involvement in early synaptic dysfunction (Vukojicic et al., 2019). Specifically, microglia inappropriately clear excitatory proprioceptive synapses from motor neurons long before actual motor neuron death occurs. This pathological synaptic stripping is driven by the classical complement cascade, whereby C1q localizes to these synapses, tagging them for microglial engulfment (Vukojicic et al., 2019). Pharmacological or genetic inhibition of the complement pathway prevents this synapse loss and improves motor function, positioning microglia and neuroimmune signaling as primary drivers of circuit dysfunction in SMA rather than mere secondary responders (Vukojicic et al., 2019).

### 7.3 Systemic metabolic defects: pancreatic and hepatic pathology

The multi-systemic nature of SMA is perhaps most profoundly evident in the metabolic disruptions observed in both patients and animal models. SMN deficiency directly impairs systemic glucose and lipid metabolism, creating a metabolic crisis that operates independently of motor neuron degeneration. Research has demonstrated that SMN depletion leads to severe pancreatic defects, including abnormal islet cell development and altered hormone secretion (Bowerman et al., 2012). Specifically, SMA models exhibit fasting hyperglycemia and hyperglucagonemia, which collectively drive glucose intolerance and impaired overall glucose homeostasis (Bowerman et al., 2012).

The liver is similarly intrinsically vulnerable to SMN loss. Liver-specific depletion of SMN causes significant peripheral tissue pathology and disrupts broader metabolic homeostasis (Alves de Almeida et al., 2025). These solid organ defects are mirrored in patient biofluids, where multi-omics profiling has revealed widespread alterations in lipid metabolism and fatty acid oxidation. Consequently, patients with SMA frequently present with metabolic acidosis and dyslipidemia, underscoring the necessity for comprehensive, multi-disciplinary metabolic care alongside emerging SMN-restoring genetic therapies.

## 8 Gene dosage sensitivity and the overexpression paradox

The delicate balance of SMN protein levels is underscored by a critical gene dosage sensitivity and an overexpression paradox. While SMN deficiency is the established cause of SMA, recent genetic and therapeutic insights reveal that an excess of SMN can also disrupt sensory-motor homeostasis.

### 8.1 Natural variation in human populations

High *SMN1* copy numbers, defined as having 3, 4, or even 5 copies of the gene, are found in healthy individuals and vary significantly by geographic ancestry. Studies have shown that sub-Saharan African populations are significantly more likely to have 3 or more copies of *SMN1* compared to populations of European or Asian descent (Hendrickson et al., 2009). Interestingly, human cell lines from healthy individuals with high *SMN1* copy numbers show that extra genetic copies do not necessarily translate to increased SMN mRNA or protein levels (Hendrickson et al., 2009). Researchers hypothesize that the body may employ feedback regulation mechanisms or epigenetic limits to tightly control physiological SMN protein levels, regardless of how many *SMN1* gene copies are present.

### 8.2 Association with adult-onset motor neuron diseases

While healthy individuals can carry high *SMN1* copy numbers, specific *SMN1* duplications have been investigated as a genetic risk factor for certain adult-onset lower motor neuron diseases. Multiple studies have found that *SMN1* duplications are significantly more frequent in patients with sporadic progressive muscular atrophy (PMA) than in healthy controls (Bos et al., 2021). Conversely, highly powered whole-genome sequencing studies have concluded that there is no association between *SMN1* copy numbers and amyotrophic lateral sclerosis (ALS) risk or disease severity (Moisse et al., 2021). Notably, when examining the symptomatology of SMA patients with 3 versus 2 copies of *SMN1*, 3 copies were associated with earlier disease onset, absence of upper motor neuron symptoms, and longer survival, with patients surviving for decades (Bos et al., 2021). At the molecular and cellular levels, it is hypothesized that duplications result in excess SMN protein, leading to upregulation and negatively impacting lower motor neurons by disrupting regulatory sequences (Bos et al., 2021).

### 8.3 Mechanisms of endogenous overexpression risk

To explain why *SMN1* duplications might increase the risk for diseases like PMA, researchers propose two main hypotheses. First, the duplicated *SMN1* genes might actually be dysfunctional haplotypes, reflecting a relative SMN protein deficiency in vulnerable motor neurons. Second, if the duplications do lead to full-length SMN transcription, an excess of SMN protein could alter the delicate stoichiometry required for its normal interactions with other cellular proteins. Because SMN is a scaffolding protein that forms large multimeric complexes, a relative abundance of SMN could disrupt crucial protein complex dynamics, eventually causing neurotoxicity.

### 8.4 Iatrogenic overexpression and the toxicity mechanism of AAV9 gene therapy

This stoichiometric disruption mirrors the potential toxic gain of function seen in modern SMA therapeutics (Rossoll and Singh, 2022). Current therapies operate through distinct mechanisms to restore SMN levels, resulting in markedly different long-term expression profiles (Shorrock and Gillingwater, 2016). Splicing modifiers like nusinersen rely on the endogenous *SMN2* gene, inherently limiting SMN induction to a natural physiological ceiling (Finkel et al., 2017). In contrast, AAV9-mediated gene replacement therapy (onasemnogene abeparvovec) introduces a transcription-competent *SMN1* cDNA driven by a constitutive promoter, creating the potential for lifelong SMN expression that far exceeds normal physiological levels (Mendell et al., 2017).

Preclinical studies in animal models have uncovered a dose-dependent, late-onset sensory-motor toxicity following AAV9-mediated SMN overexpression (Van Alstyne et al., 2021). Because AAV9 vectors possess strong tropism for post-mitotic motor and sensory neurons, viral episomes do not dilute over time, leading to massive localized SMN accumulation. This introduces a critical paradox: if monomeric SMN is normally targeted for rapid proteasomal degradation, how does it accumulate to thresholds sufficient to form dominant-negative aggregates?

The answer lies in the kinetics of oligomerization and clearance under conditions where transgene-driven SMN production exceeds the buffering capacity of the endogenous proteostasis machinery. Under physiological conditions, full-length SMN exists in two biologically distinct stability pools (Burnett et al., 2009; Custer et al., 2013). Oligomeric SMN self-associates via its YG-box domain into dimers, tetramers, and octamers, sterically occluding an internal phosphodegron and shielding the complex from degradation (Martin et al., 2012). Conversely, monomeric SMN retains an exposed phosphodegron: phosphorylation of S270 by GSK3β primes it for SCFSlmb recognition, triggering K48-linked polyubiquitination and proteasomal clearance (Gray et al., 2018). When transgene-driven synthesis outpaces endogenous production, the influx of monomeric SMN saturates the finite throughput of the SCFSlmb complex, leaving a fraction of the monomeric pool to escape degradation and progressively form large, insoluble cytoplasmic aggregates (Van Alstyne et al., 2021).

These aggregates exert a potent dominant-negative effect. Unassembled monomeric SMN retains a functional Tudor domain capable of binding Sm proteins with high affinity (Sprangers et al., 2003), sequestering them away from productive snRNP assembly by fully oligomeric complexes. The resulting impairment of snRNP biogenesis mimics the molecular deficit of SMN deficiency and produces DRG neuropathy, localized inflammation, and selective deafferentation of motor neurons via loss of excitatory proprioceptive synapses (Van Alstyne et al., 2021).

This dual-edged pathology demonstrates that the safe therapeutic window is defined not merely by total SMN abundance, but by the kinetic balance between SMN production and the buffering capacity of two parallel protective systems: SCFSlmb-mediated degradation of free monomers and Gemin2-mediated incorporation of nascent SMN into functional complexes (Gray et al., 2018; Zhang et al., 2011). Strategies to widen this window include limiting transgene expression via tunable switches or co-delivering assembly scaffolds such as Gemin2, though the latter is constrained by the approximately 4.7 kb packaging limit of AAV vectors. Prospective monitoring of biomarkers reflecting SMN complex assembly stoichiometry, rather than absolute protein abundance alone, will be essential to safely navigating this therapeutic window (Van Alstyne et al., 2021).

### 8.5 Downstream transcriptome and immune alterations

The sequestration of Sm proteins by overexpressed SMN leads to widespread dysregulation of RNA processing in the DRG, triggering substantial downstream transcriptome abnormalities. Gene ontology analyses of DRGs subjected to long-term SMN overexpression reveal a strong induction of neuroinflammation and innate immune response pathways. Strikingly, this includes the significant upregulation of the C1q complement complex. As described in Section 7.2, C1q-mediated microglial synaptic stripping is a primary driver of early circuit dysfunction in SMA deficiency models; the fact that iatrogenic SMN overexpression triggers the same complement cascade suggests that both extremes of SMN dysregulation converge on a shared neuroinflammatory endpoint (Van Alstyne et al., 2021).

### 8.6 Implications for SMA therapeutics

The discovery that SMN overexpression exerts a toxic gain of function has profound clinical implications for the long-term safety of SMA gene therapy. While AAV9-SMN is life-saving for infants with severe SMA type 1, the risk of late-onset neurotoxicity warrants careful monitoring, particularly if applied to milder SMA patients who already possess higher endogenous *SMN2* copy numbers (Van Alstyne et al., 2021). These findings highlight the risks of uncontrolled, long-lasting transgene expression and underscore the need for next-generation gene therapy vectors equipped with optimized promoters, microRNA target sequences for detargeting, or embedded tunable expression switches to maintain SMN levels within a safe therapeutic window.

## 9 Monitoring SMN levels: CNS vs. peripheral tissues

Accurate quantification of SMN is vital for monitoring therapeutic efficacy. While directly measuring central nervous system (CNS) levels in patients is challenging, peripheral tissues serve as highly effective biomarkers. SMN protein concentrations can be reliably measured using electrochemiluminescence (ECL) immunoassays and ELISAs in whole blood and peripheral blood mononuclear cells (PBMCs) (Steinkellner et al., 2015; Kobayashi et al., 2011). Buccal swabs and skin fibroblasts similarly provide excellent, non-invasive surrogate tissues for analysis. The clinical utility of these peripheral measurements is strongly supported by preclinical models, which demonstrate that SMN levels in the peripheral blood correlate significantly with those found in the brain and spinal cord. Beyond direct protein quantification, clinicians also rely on functional readouts to assess therapeutic impact. Diagnostic imaging and testing, such as high-resolution ultrasonography (HRUS) and electromyography (EMG), are routinely utilized to monitor the downstream electrophysiological and structural rescue of motor units over time (Wurster et al., 2019; Arnold et al., 2015).

SMN is also transported between cells in extracellular vesicles (EVs). Recent studies demonstrate that the SMN protein is naturally released from cells within exosomes, and the quantity of SMN protein contained in these serum-derived vesicles directly correlates with the disease genotype in both human patients and spinal muscular atrophy mouse models (Nash et al., 2017). Measuring SMN levels in serum-derived extracellular vesicles therefore represents a promising, minimally invasive diagnostic biomarker (Nash et al., 2017). Beyond the SMN protein itself, recent advances have highlighted the biomarker potential of specific SMN RNA species encapsulated within these vesicles. For example, the expression of the full-length SMN transcript in serum extracellular vesicles significantly increases in SMA type 2 patients following nusinersen treatment, mirroring the therapeutic response (Trifunov et al., 2023). Notably, specific circular RNAs derived from the SMN gene, such as SMN circ4-2b-3, are also detectable in patient exosomes and correlate with improved motor outcomes, helping to identify strong responders to antisense oligonucleotide therapies (Guerra et al., 2025).

Clinical heterogeneity in response to *SMN2*-targeting therapies reflects not only differences in *SMN2* copy number but also patient-specific variation in the baseline splicing regulatory environment and the capacity of individual cells to respond to pharmacological intervention. Analysis of 45 primary SMA patient fibroblast lines treated with nusinersen and risdiplam demonstrates substantial inter-patient variability in SMN protein induction that cannot be fully predicted by genotype alone (Signoria et al., 2024). These findings have direct implications for personalized treatment selection and highlight the need for ex vivo cell-based functional assays as companion diagnostics to guide therapeutic matching, particularly as combination regimens incorporating multiple SMN-modifying agents are developed.

## 10 Therapeutic targeting: combination therapies and novel modifiers

While current therapies successfully increase SMN production, achieving complete phenotypic rescue in older or symptomatic patients remains a challenge. As detailed in Section 8, the risks of both deficiency and overexpression mean that effective therapy must do more than simply maximize SMN levels; it must maintain them within a defined physiological range (Bowerman et al., 2012). Pairing an upstream production modifier with downstream protein stabilizers or novel genetic targets offers a strategy to increase steady-state SMN levels while reducing reliance on massive transgene expression (Signoria et al., 2023). Moreover, a critical and underappreciated dimension of small-molecule splicing modifiers is their target breadth beyond *SMN2*. Comprehensive transcriptomic analysis has revealed that *SMN2*-directed splicing modulators alter exon inclusion in hundreds of pre-mRNA targets genome-wide, with only a subset of these off-target splicing events being clinically tolerable (Ottesen et al., 2023). This promiscuity arises because the RNA sequence features recognized by these compounds are not unique to the *SMN2* exon 7 context. The therapeutic implication is that maximizing *SMN2* exon 7 inclusion through dose escalation of small-molecule splicing modifiers may not be straightforwardly achievable without incurring proportionally greater off-target splicing toxicity, reinforcing the case for combination strategies that achieve adequate SMN levels at submaximal individual drug doses.

### 10.1 Modulating the ubiquitin-proteasome system

Broad-spectrum UPS inhibitors (e.g., Bortezomib) prevent SMN degradation but carry risks of cellular toxicity. A safer alternative is the targeted pharmacological or genetic inhibition of specific E3 ligases, such as Mib1 (Kwon et al., 2013). Beyond E3 ligase inhibition, developing small molecules to activate USP9X or utilizing emerging deubiquitinase targeting chimeras (DUBTACs) could intentionally recruit USP9X directly to the SMN protein, actively protecting it from destruction.

### 10.2 The GEMIN proteins: from scaffolds to disease-modifying targets

Historically, the Gemin proteins (Gemin2 through Gemin8, along with Unrip) have been viewed primarily as passive structural scaffolds. However, accumulating evidence demonstrates that Gemins actively modulate the expression, stability, and overall activity of the SMN complex (Feng et al., 2005). This shift in perspective positions the GEMINs as potent, independent disease-modifying targets for SMA.

Rather than simply holding the complex together, several Gemins provide critical enzymatic and regulatory functions. Gemin3 is a DEAD-box RNA helicase that utilizes ATP to facilitate the structural RNA rearrangements necessary during snRNP biogenesis (Charroux et al., 1999). Gemin5, the largest member of the complex, functions as the primary RNA-binding protein. Through its highly conserved WD40 repeats and tetratricopeptide repeat domains, Gemin5 recognizes the Sm-site of pre-snRNAs and delivers them directly to the SMN complex (Battle et al., 2006). Notably, Gemin5 independently regulates selective mRNA translation and ribosome binding, and mutations in its sequence have been linked to distinct neurodevelopmental syndromes. Other components, such as Gemin4 and Gemin8, are indispensable for the proper architectural integration of the complex and its localization to Cajal bodies and nucleoli (Carissimi et al., 2006; Charroux et al., 2000).

The concept of targeting GEMINs therapeutically relies on maximizing the efficiency of the residual SMN protein present in all SMA patients. Upregulating specific Gemin proteins could theoretically stabilize scarce SMN molecules or boost the catalytic turnover rate of remaining SMN complexes. By enhancing the activity of the SMN complex downstream of *SMN1* transcription, GEMIN-targeted therapies could bypass the limitations and potential toxicities associated with massive SMN overexpression.

### 10.3 Novel genetic modifiers of spinal muscular atrophy

In the search for new SMN-enhancing strategies and SMN-independent targets, the identification of genetic modifiers has opened entirely new therapeutic avenues. A pivotal breakthrough came from the study of rare, fully asymptomatic individuals who carry homozygous deletions of the *SMN1* gene but remain disease-free due to the altered expression of protective modifier genes. The first and most prominent of these to be identified was plastin 3 (PLS3), an actin-bundling protein. The overexpression of PLS3 rescues axonal outgrowth and neuromuscular junction pathology in models of spinal muscular atrophy by restoring impaired endocytosis and actin dynamics, operating completely independently of SMN protein levels (Oprea et al., 2008; Hosseinibarkooie et al., 2016). Building upon the endocytic rescue pathway, coronin 1C (CORO1C) was subsequently identified as an interacting partner of PLS3 that similarly confers protection against motor neuron degeneration when overexpressed (Hosseinibarkooie et al., 2016). Conversely, the reduction of certain genes has also been shown to be highly protective. For instance, the targeted knockdown of the calcium-sensor protein neurocalcin delta (NCALD) acts as a powerful negative modifier, significantly ameliorating the spinal muscular atrophy phenotype across multiple species by restoring impaired synaptic vesicle endocytosis (Riessland et al., 2017).

Alongside these SMN-independent protective pathways, innovative screening strategies have identified diverse classes of modifiers that directly govern SMN expression and stability. Pivotal work in high-throughput compound screening previously established that the disease-modifying *SMN2* gene could be exploited to identify diverse small-molecule modulators that elevate full-length SMN protein levels, paving the way for targeted therapeutic discovery (Jarecki et al., 2005). More recently, high-throughput genome-wide RNAi screens have discovered modifiers heavily involved in RNA processing, polyadenylation, and mRNA nuclear export, specifically WDR33, CPSF1, and THOC3, whose targeted knockdown significantly increases SMN protein expression in patient cells (McCormack et al., 2021). In a complementary approach, utilizing a human SMN2-GFP reporter system, researchers discovered that specific cysteine proteases, including CAPN1, CAPN7, CTSB, and CTSL, mediate the degradation of SMN proteins. Pharmacological inhibition of these proteases successfully stabilizes SMN and mitigates mitochondriopathy and neuropathy in patient-derived motor neurons, presenting a highly viable therapeutic strategy (Wang et al., 2019).

Large-scale genetic screens in model organisms like *Drosophila* have also been instrumental in defining the broader SMN interactome, identifying hundreds of candidate genes that alter SMN-dependent phenotypes *in vivo* (Sen et al., 2013). Such genomic approaches have further elucidated SMN’s critical role in the targeted trafficking and post-transcriptional regulation of specific mRNAs, reinforcing the importance of localized translation in disease pathogenesis (Aquilina and Cauchi, 2018). Finally, specific mutant variants of synaptic chaperones can exert profound protective effects independent of baseline gene expression. For example, the introduction of a specific *Hspa8* variant (Hspa8G470R) in SMA mouse models remarkably suppressed the disease, increasing lifespan over tenfold (Kim et al., 2023). Mechanistically, this variant restored synaptic vesicular SNARE complex formation, which is typically severely disrupted in SMA motor neurons, highlighting the vast therapeutic potential of targeting synaptic assembly machinery alongside canonical SMN restoration (Kim et al., 2023).

## 11 Conclusion

The survival motor neuron protein is far more than a canonical splicing factor. It is a dynamic, multi-compartmental assembly factor whose precise regulation is essential not only for motor neuron survival but for the integrity of skeletal muscle, glial cells, and metabolic organs. As this review has outlined, the pathogenesis of SMA is driven by an interplay of inadequate SMN production, rapid degradation of the unstable SMNΔ7 isoform, cell-autonomous dysfunction across multiple tissues, and, paradoxically, the toxicity that can arise from therapeutic overexpression. The intricate balance between E3 ligases and deubiquitinases highlights the ubiquitin-proteasome system as a critical and actionable frontier, while the complement-driven microglial stripping of synapses and the intrinsic metabolic vulnerabilities of non-neuronal tissues underscore how much of SMA pathology remains undertreated by current neuron-centric approaches.

Moving forward, the most promising therapeutic avenues will require synergistic combination strategies that pair established genetic therapies with pharmacological agents designed to stabilize residual SMN, enhance GEMIN complex activity, or engage SMN-independent protective pathways such as PLS3 and NCALD. Several important questions remain open: What is the precise physiological SMN window across different tissue types and disease stages? What are the barriers to implementing dual-modality therapy in symptomatic patients? And how can next-generation AAV vectors be designed to provide durable, titratable expression without the risks of aggregate-driven toxicity? Addressing these questions will be essential to translating the remarkable mechanistic insights reviewed here into therapies that are both safe and sufficient for patients across the full spectrum of SMA severity.
